# Disseminated Blastomycosis Infection in an Immunocompetent Patient

**DOI:** 10.7759/cureus.104679

**Published:** 2026-03-04

**Authors:** Mario Tavakoli, Gautham Chitturu

**Affiliations:** 1 Medicine, University of Illinois Chicago, Chicago, USA; 2 Internal Medicine, University of Massachusetts, Worcester, USA

**Keywords:** disseminated blastomycosis, general internal medicine, infectious disease diagnosis, internal medicine, orthopedic infections

## Abstract

Blastomycosis is a systemic pyogranulomatous infection that primarily involves the lungs and often results in asymptomatic lung infections. In some cases, however, the fungus can lead to acute or chronic pneumonia. Rarely, hematogenous spread can occur, with the most common extrapulmonary manifestations including skin, bone, and genitourinary involvement. We present a case of a 20-year-old male patient with no significant past medical history who presented with lower extremity pain and difficulty walking. After an extensive work-up, the patient was found to have disseminated blastomycosis with bone and skin involvement. The patient’s age and immunocompetent status make this case particularly puzzling and highlight the need for healthcare providers to remain vigilant regarding fungal diseases, especially in endemic areas.

## Introduction

Blastomycosis is a systemic pyogranulomatous infection that primarily involves infection of the lungs. The fungus is inoculated in humans through inhalation of conidia through the air and is typically found in areas of moist soil rich in decomposing organic material. Its geographic preponderance mainly exists in North America, with most infections arising in the states surrounding the Great Lakes and those bordering the Ohio and Mississippi river basins [1]. Infections from blastomycosis often result in asymptomatic lung infections, although some can transition to acute or chronic pneumonia [2]. In some cases, however, hematogenous spread can occur with the most common extrapulmonary manifestations, including skin, bone, and genitourinary system involvement [3].

In a 1997 study, 326 patients with blastomycosis were evaluated to determine the most common clinical manifestations of the disease [4]. It was reported that 91% of patients had lung involvement, resulting in either asymptomatic disease or pneumonia, with 17% of patients having multisystem organ involvement [4]. While the pathogenesis of disseminated blastomycosis is not fully understood, many forms of extrapulmonary disease occur after the primary pulmonary infection has resolved. This reactivation of disease is common in immunocompromised patients, especially those taking tumor necrosis factor-alpha (TNF-α) inhibitors or corticosteroids for comorbid conditions [2]. However, the incidence of disseminated blastomycosis in immunocompetent individuals is not well understood. We present a case of a 20-year-old male patient with no significant past medical history who presented with lower extremity pain and difficulty walking and was found to have disseminated blastomycosis with skin and bone involvement.

## Case presentation

**Figure 1:**
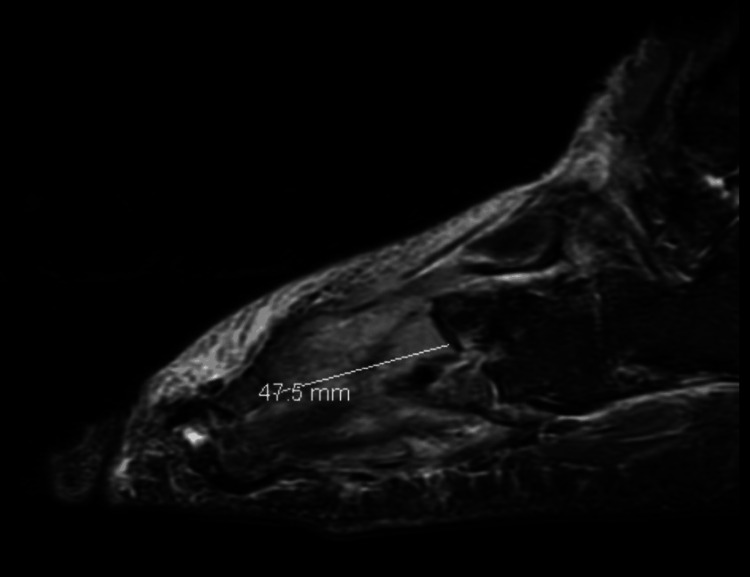
Initial non-contrast foot MRI revealed an ill-defined soft tissue mass involving the base of the third metatarsal, concerning for malignancy or infection. A 4.75 cm ill-defined soft tissue mass was noted involving the base of the third metatarsal. MRI: magnetic resonance imaging

A 20-year-old male patient with no past medical history presented to the emergency department (ED) with two months of right forefoot bruising along with difficulty with ambulation and weight bearing. The pain had progressed to the point that he was unable to walk long distances without crutches. One month after symptom onset, the patient was seen by a podiatrist at an outside facility, who determined that the pain was likely due to tendonitis. Magnetic resonance imaging (MRI) at the time revealed a soft tissue mass in his foot that was concerning for malignancy or an infection (Figure 1). He was started on ciprofloxacin and clindamycin and was referred to orthopedic oncology. Although the patient was compliant with his medications, his bruising progressed to ulceration with increased pain one month later. The wound subsequently burst, prompting him to be evaluated at an outside ED.

**Figure 2:**
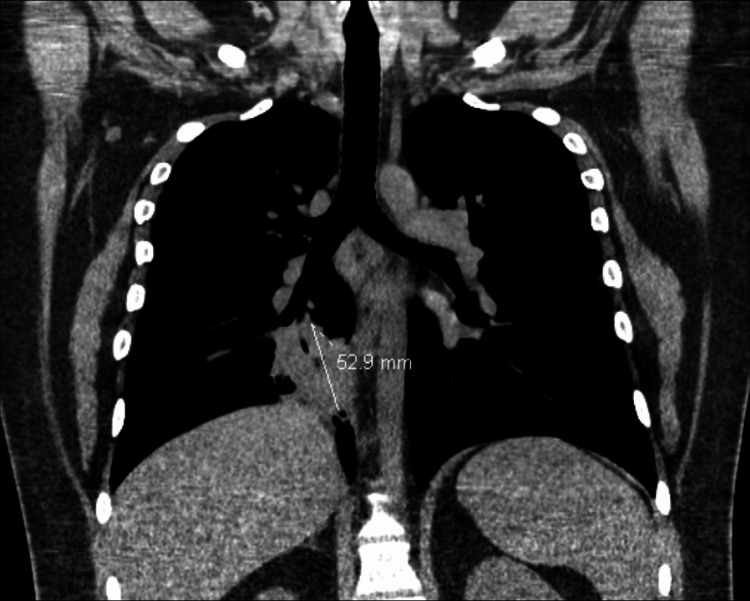
CT chest revealed a moderate-sized focal area of consolidation in the right lung with an intramammary lymph node. A 5.29 cm focal area of consolidation was noted in the right lung. CT: computed tomography

The patient presented to the ED and was afebrile and hemodynamically stable. His major complaint was his intense foot pain, but he also reported having a cough with occasional blood-streaked sputum for the past month. Blood cultures obtained were found to be negative. Wound cultures were positive for *Staphylococcus epidermidis*, and fungal cultures were pending. Notably, the laboratory results included an erythrocyte sedimentation rate (ESR) of 48 mm/hr (normal range: <10 mm/hr) and a C-reactive protein (CRP) level of 16.9 mg/L (normal range: <8.0 mg/L). A foot MRI was obtained, and it was concerning for osteomyelitis vs. malignancy. Chest computed tomography (CT) revealed a focal area of consolidation in the right lung with intramammary lymph nodes (Figure 2). He was initially started on Zosyn but was transitioned to cefazolin. The patient was scheduled for wound debridement with podiatry, but the providers instead decided to transfer him to an institution with orthopedic oncology. 

**Figure 3:**
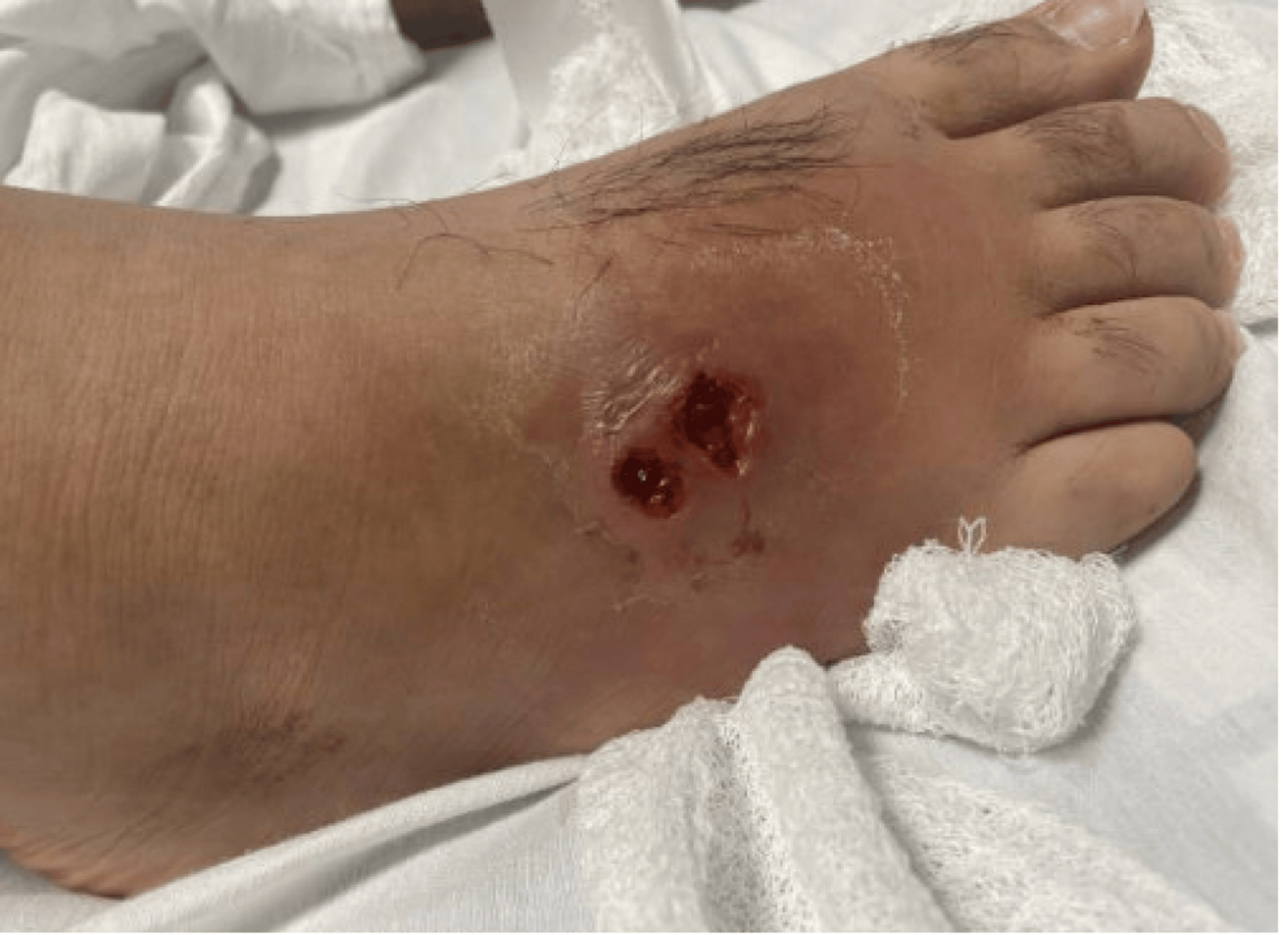
An erythematous ulcerated wound was observed on the right lateral forefoot without any drainage.

Upon further investigation, the patient also endorsed an unintentional 30-pound weight loss in the last three months, along with night sweats that he had never experienced before. The patient denied any fever, chills, chest pain, shortness of breath, nausea, vomiting, diarrhea, constipation, dysuria, or hematuria. He confirmed that he had no past medical or surgical history, was a nonsmoker, and did not drink alcohol or use recreational drugs. The patient reported a history of an unknown blood cancer in his maternal grandfather, but no other pertinent family history. The patient’s vital signs were stable, and he was saturating well on room air. He had a body mass index (BMI) of 33 kg/m^2 ^and did not appear cachectic. An erythematous ulcerated wound was observed on the right lateral forefoot without any drainage (Figure 3).

**Figure 4:**
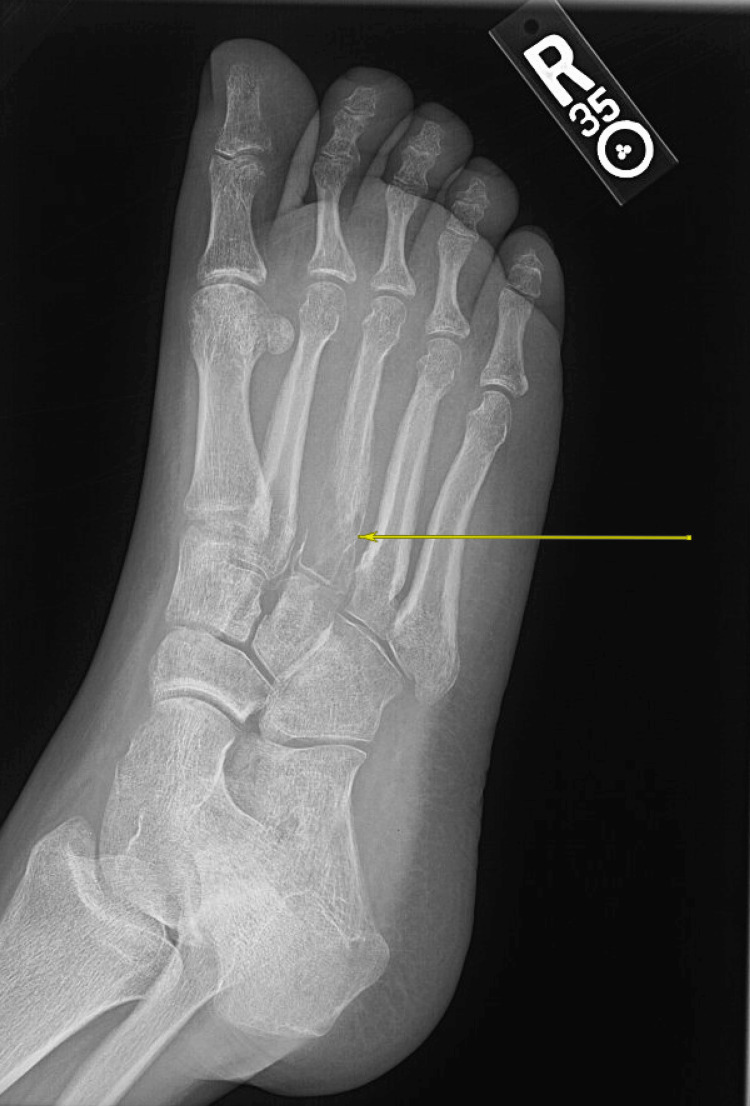
Foot X-ray demonstrated focal osteopenia with extensive resorptive changes of the proximal third metatarsal with periosteal thickening and overlying soft tissue swelling, most concerning for osteomyelitis. The arrow indicates the focal area of osteopenia of the proximal third metatarsal.

Additionally, the patient did not have an elevated white cell count, and his basic metabolic panel (BMP) was normal. However, his foot X-ray was concerning for osteomyelitis and revealed focal osteopenia with extensive resorptive changes in the proximal third metatarsal bone with periosteal reactions and thickening of the overlying soft tissue (Figure 4).

**Figure 5:**
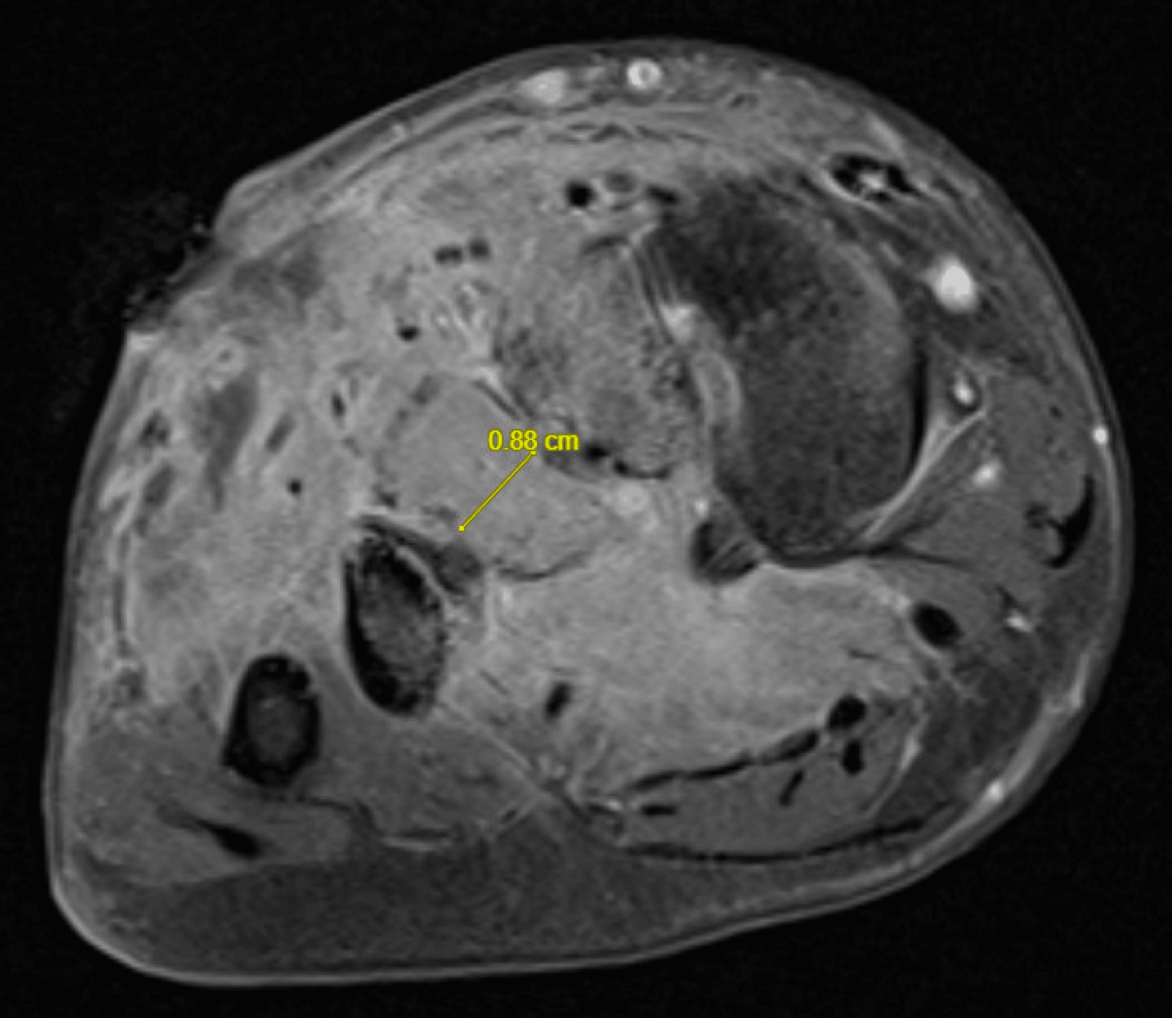
Repeat foot MRI revealed osteomyelitis of the third metatarsal with involvement of the adjacent second and fourth metatarsal bases, diffuse cellulitis, and multiple tiny foci of bone edema in the forefoot and midfoot. A 0.88 cm area of inflammation was noted, concerning for osteomyelitis of the third metatarsal with diffuse overlying cellulitis.

The patient was broadened to vancomycin and Zosyn for methicillin-resistant *Staphylococcus aureus *(MRSA) coverage. A subsequent foot MRI revealed osteomyelitis of the third metatarsal bone with involvement of the adjacent second and fourth metatarsals, similar to the MRI obtained at the outside hospital. There was associated skin ulceration with an underlying early abscess extending to the third metatarsal, which was new compared to prior imaging. Diffuse cellulitis and multiple tiny foci of bone edema in the forefoot and midfoot were observed, which is unusual for osteomyelitis but could represent possible diffuse osteopenia or reflex sympathetic osteodystrophy resulting from a prior injury (Figure 5).

An infectious disease (ID) consultation revealed that the patient had also developed small erythematous nodules on his left thigh, left ear, and back over the past three months. Their findings elicited a high suspicion for disseminated blastomycosis due to pulmonary, cutaneous, and osteoarticular involvement and a lack of response to antibacterial agents. Subsequently, the ID team recommended stopping broad-spectrum antibiotics and starting itraconazole and amphotericin. They recommended sending further studies, including human immunodeficiency virus (HIV), tuberculosis, gonorrhea, and chlamydia, which were all negative. Their team also requested that dermatology obtain skin biopsies of the right foot lesion for bacterial cultures and stains to confirm their suspected diagnosis. A subsequent urine sample was positive for blastomyces antigens, and foot biopsies confirmed the diagnosis. The punch biopsy revealed diffuse dermal and subcutaneous suppurative granulomatous inflammation with evidence of broad budding yeast organisms and numerous scattered multinucleated giant cells. Gram stain showed scattered bacterial colonies of gram-positive cocci in chains and pairs, while the acid-fast bacilli (AFB) stain was negative. The Grocott’s methenamine silver stain for fungus (GMS-F) and periodic acid-Schiff stain for fungus (PAS-F) revealed scattered nonbudding single yeast forms and broad-based budding yeast forms most consistent with blastomycosis.

## Discussion

This case represents findings that were initially unsuspected of a potential fungal infection. Upon further workup and evaluation, however, the patient’s history, symptoms, imaging, and pathology results gave way to a clear diagnostic picture of disseminated blastomycosis. Various factors, such as the patient’s age, initial presenting symptom of a foot ulcer, and immunocompetent status, made it difficult to arrive at the correct diagnosis earlier in the disease course. In this discussion, we aim to elucidate how these factors serve as focal pieces of his overall clinical picture.

This case involved a 20-year-old male patient who, for statistical purposes, falls well outside the typical patient population. A review from the State Inpatient Database of the United States Agency for Healthcare Research and Quality evaluated the incidence of blastomycosis infection in 46 states. They determined that the median age for disseminated blastomycosis resulting in hospitalization was 53 years [5]. While this dataset can be useful from a statistical standpoint, its clinical significance is limited. Although past studies suggested that patients with occupations in agriculture and forestry had higher incidence rates of infection, most recently reported outbreaks indicate no sex, age, occupation, or seasonal predilection for blastomycosis [6]. The most important risk factor, in fact, is extended exposure to soil [7]. While we know that this patient is from the greater Chicago area and thus is included in the endemic population, his occupation or experience with soil remains unknown. Next, the patient’s initial foot ulcer presentation and difficulty with bearing weight pointed toward isolated cellulitis, osteomyelitis, or malignancy. Before reaching any conclusions, it is necessary to discuss the general pathogenesis of blastomycosis. The incubation period for blastomycosis is approximately 3-6 weeks, and the fungus commonly manifests as a pulmonary infection [3]. A 1997 study evaluated 326 patients with blastomycosis infection to determine the most common clinical manifestations of the disease and reported that 91% of patients had lung involvement, resulting in either asymptomatic disease or pneumonia [4]. An extensive history revealed that our patient had, in fact, been experiencing a productive cough with intermittent bloody sputum for one month prior to his initial presentation. Chest CT revealed nodularity and focal opacity in the right lung, and chest X-ray revealed some level of consolidation in the corresponding area. Other case reports indicate that chest X-rays may reveal alveolar infiltrates or a mass lesion, whereas chest CT may reveal nodules or consolidations with or without cavitation [8]. While it is apparent that these findings are nonspecific, they certainly do not rule out fungal infection or the presence of a chronic lung infection. This pattern is a hallmark feature of blastomycosis, and a history of prior lung infection with new systemic manifestations, including skin and bone involvement, should be further explored for disseminated blastomycosis.

In the 1997 study of 326 patients with blastomycosis, 18% had concomitant skin involvement, whereas only 4% had bone involvement [4]. For systemic fungal infection to remain a consideration, one must evaluate skin and bone involvement in the context of a lung infection due to potential hematogenous dissemination of an endemic fungus. Although the characteristic cutaneous findings of blastomycosis infection are described as raised verrucous lesions with irregular borders, ulcerative lesions that bleed easily and have well-demarcated, heaped borders may also occur, possibly mimicking squamous cell carcinoma [1]. The patient reported prior episodes of skin lesions throughout his body, which were noted on examination as crusted-over, raised lesions. Typically, bone involvement in disseminated blastomycosis manifests as osteomyelitis. Among patients hospitalized for multisystem disease, only 25% of patients have bone involvement [9]. This is characterized mainly as a well-circumscribed osteolytic lesion, most commonly found on the lower limbs or axial skeleton [10]. In this patient, the lesion was present on the third metatarsal and the adjacent second and fourth metatarsals. The hematogenous spread of fungal disease most commonly presents as multiorgan involvement, as discussed previously. This patient’s immunocompetent status complicates assessment of his risk for dissemination and further represents a conundrum in clinical decision-making. For other endemic mycoses, such as histoplasmosis and coccidioidomycosis, immunosuppression is associated with an increased risk for dissemination. For blastomycosis, however, recent studies demonstrate no association between immune status and dissemination [11]. This has important implications in the present case, as it increases the risk of a silent prodromal course of infection. Therefore, the patient’s immune status does not rule out a diagnosis of disseminated blastomycosis.

## Conclusions

Overall, the patient’s presentation of symptoms, with confirmed biopsy results, revealed disseminated blastomycosis. This patient’s atypical presentation made it difficult to pinpoint the diagnosis sooner, and there are several major takeaways that can be gleaned from this case. First, patients who live in regions endemic for blastomycosis should be tested for the fungus if they present with community-acquired pneumonia (CAP) of unknown etiology that does not respond to empiric antibiotics. Second, patients who present with CAP and characteristic skin lesions should be evaluated for a systemic fungal disease, especially if they are from an endemic region. Finally, any patient with signs of extrapulmonary blastomycosis, negative bacterial cultures, and no response to initial antibiotic therapy should also be evaluated for a fungal infection.

In Illinois, blastomycosis infection should be considered with extra caution due to proximity to endemic regions. Disseminated blastomycosis has a high mortality rate, and because it does not have a preponderance for immunocompromised patients, infection can be clinically silent in many patients. Thoroughly considering blastomycosis when practicing in endemic regions is important when working up infectious presentations.

## References

[1] Saccente M, Woods GL (2010). Clinical and laboratory update on blastomycosis. Clin Microbiol Rev.

[2] Bradsher RW, Chapman SW, Pappas PG (2003). Blastomycosis. Infect Dis Clin North Am.

[3] Azar MM, Assi R, Relich RF, Schmitt BH, Norris S, Wheat LJ, Hage CA (2015). Blastomycosis in Indiana: clinical and epidemiologic patterns of disease gleaned from a multicenter retrospective study. Chest.

[4] Chapman SW, Lin AC, Hendricks KA, Nolan RL, Currier MM, Morris KR, Turner HR (1997). Endemic blastomycosis in Mississippi: epidemiological and clinical studies. Semin Respir Infect.

[5] Seitz AE, Younes N, Steiner CA, Prevots DR (2014). Incidence and trends of blastomycosis-associated hospitalizations in the United States. PLoS One.

[6] Pappas PG, Threlkeld MG, Bedsole GD, Cleveland KO, Gelfand MS, Dismukes WE (1993). Blastomycosis in immunocompromised patients. Medicine (Baltimore).

[7] Klein BS, Vergeront JM, Davis JP (1986). Epidemiologic aspects of blastomycosis, the enigmatic systemic mycosis. Semin Respir Infect.

[8] Sheflin JR, Campbell JA, Thompson GP (1990). Pulmonary blastomycosis: findings on chest radiographs in 63 patients. AJR Am J Roentgenol.

[9] Moore RM, Green NE (1982). Blastomycosis of bone. A report of six cases. J Bone Joint Surg Am.

[10] Vaaler AK, Bradsher RW, Davies SF (1990). Evidence of subclinical blastomycosis in forestry workers in northern Minnesota and northern Wisconsin. Am J Med.

[11] McBride JA, Sterkel AK, Matkovic E, Broman AT, Gibbons-Burgener SN, Gauthier GM (2021). Clinical manifestations and outcomes in immunocompetent and immunocompromised patients with blastomycosis. Clin Infect Dis.

